# MXene-based all-solid flexible electrochromic microsupercapacitor

**DOI:** 10.1038/s41378-024-00720-6

**Published:** 2024-06-25

**Authors:** Shanlu Guo, Ruihe Zhu, Jingwei Chen, Weilin Liu, Yuxiang Zhang, Jianmin Li, Haizeng Li

**Affiliations:** 1 College of Electronic and Optical Engineering & College of Flexible Electronics (Future Technology), Nanjing University of Posts and Telecommunications, Nanjing, 210023 China; 2 School of Materials Science and Engineering, Ocean University of China, Qingdao, 266100 China; 3 Optics and Thermal Radiation Research Center, Institute of Frontier and Interdisciplinary Science, Shandong University, Qingdao, 266237 China; 4 Shenzhen Research Institute of Shandong University, Shenzhen, 518000 China; 5 State Key Laboratory of Featured Metal Materials and Life Cycle Safety for Composite Structures, Guangxi University, Nanning, 530004 China

**Keywords:** Micro-optics, Micro-optics

## Abstract

With the increasing demand for multifunctional optoelectronic devices, flexible electrochromic energy storage devices are being widely recognized as promising platforms for diverse applications. However, simultaneously achieving high capacitance, fast color switching and large optical modulation range is very challenging. In this study, the MXene-based flexible in-plane microsupercapacitor was fabricated via a mask-assisted spray coating approach. By adding electrochromic ethyl viologen dibromide (EVB) into the electrolyte, the device showed a reversible color change during the charge/discharge process. Due to the high electronic conductivity of the MXene flakes and the fast response kinetics of EVB, the device exhibited a fast coloration/bleaching time of 2.6 s/2.5 s, a large optical contrast of 60%, and exceptional coloration efficiency. In addition, EVB acted as a redox additive to reinforce the energy storage performance; as a result, the working voltage window of the Ti_3_C_2_-based symmetric aqueous microsupercapacitor was extended to 1 V. Moreover, the device had a high areal capacitance of 12.5 mF cm^−2^ with superior flexibility and mechanical stability and showed almost 100% capacitance retention after 100 bending cycles. The as-prepared device has significant potential for a wide range of applications in flexible and wearable electronics, particularly in the fields of camouflage, anticounterfeiting, and displays.

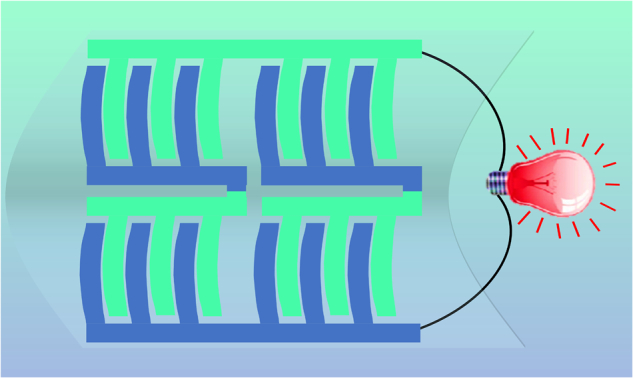

## Introduction

Flexible electrochromic energy storage devices, which exhibit synchronous color changes accompanied by charge/discharge processes, are rapidly evolving because of their potential in wearable electronics, miniaturized indicators, multifunctional devices, and human‒machine interfaces^1–8^. On the one hand, the color change and energy storage functions can work independently. On the other hand, the concomitant color change provides a visual indication of the charge/discharge states of the electrochemical process. Because of the similar device structure and working mechanism between supercapacitors and electrochromic devices, an integrated electrochromic energy storage dual-functional device is possible and can be easily achieved^9–11^.

The in-plane-configured microsupercapacitor shows better flexibility, simpler structure, and easier integration ability than conventional sandwich supercapacitors^12–15^. In addition, the planar configuration could optimize the diffusion pathway of ions among the layers of the two-dimensional (2D) materials and thus facilitate the kinetics by preventing the diffusion of ions in the direction perpendicular to the 2D flakes^16–21^. As a result, compared with traditional sandwich devices, microsupercapacitors usually exhibit better rate capability. Therefore, in the past few years, the fabrication of planar electrodes has attracted significant interest in the design of high-performance electrochromic energy storage devices.

For instance, Qin et al.^13^ reported a symmetric electrochromic microsupercapacitor (EMS) with Ag nanowire/NiO composite films as the electrodes; here, the positive electrode switched to a dark color during the charging process due to the oxidation of NiO. However, the coloration efficiency (CE) and color contrast were much less than those of the bare electrochromic devices. Moreover, the use of indium tin oxide (ITO) as the transparent conductive layer caused performance degradation during mechanical deformation; this resulted in inferior deformability for the flexible electronics. In addition, we previously fabricated a symmetric EMS with 2D MXene as the transparent conductive layer and poly(3,4-ethylenedioxythiophene) (PEDOT) as the active material^16^. Due to the good mechanical performance of both MXene and PEDOT, the device showed good flexibility. Importantly, the color contrast of the device was comparable to those of traditional electrochromic devices, with slightly compromised color-switching kinetics.

Notably, the emerging family of 2D transition metal carbides/nitrides, termed MXenes, is attractive for fabricating transparent conductive films (e.g., Ti_3_C_2_, V_2_C, and Nb_4_C_3_) due to their excellent electrical conductivity^22–25^. In addition, MXenes usually exhibit good solution processability, enabling the formation of transparent thin films on various substrates, such as glass, polyethylene terephthalate (PET), and polydimethylsiloxane (PDMS)^26–29^. Additionally, due to their surface oxygen-containing terminations (=O, ‒OH), highly conductive MXenes have strong redox activity, resulting in excellent high-rate charge transfer and energy storage capabilities^30^. Due to these properties, MXenes are attractive candidates for fast-responsive EMSs.

Herein, in this study, we fabricated in-plane interdigitated Ti_3_C_2_ MXene thin films as electrodes of microsupercapacitors using a mask-assisted spray coating approach. The Ti_3_C_2_ films served as both transparent conductive electrodes and active energy storage materials. Benefiting from the high electrical conductivity and versatile terminations of MXenes, the device exhibited high specific capacitance and good rate capability in the gel electrolyte composed of 1 M H_2_SO_4_ with polyvinyl alcohol (PVA). Additionally, electrochromic ethyl viologen dibromide (EVB) was added to the electrolyte to achieve a fast color change between deep green and blue. Due to the excellent electron acceptance ability of EVB, the device had a fast response at a coloration/bleaching time of 2.6 s/2.5 s. The as-prepared device shows promising potential for future flexible and wearable electronics with the demands of camouflage, anticounterfeiting, and displays.

## Results and discussion

The 2D Ti_3_C_2_ MXene suspensions were prepared using the previously reported minimally intensive layer delamination method; the method details are provided in the “Methods” section^31^. Briefly, the titanium aluminum carbide (Ti_3_AlC_2_) precursor was slowly added to a mixed aqueous solution of HCl/LiF, followed by stirring for 24 h. After washing and shaking several times in deionized (DI) water, the colloidal aqueous suspension of Ti_3_C_2_ MXene was collected. The morphology of the single-layer Ti_3_C_2_ sheet was imaged by scanning electron microscopy (SEM); here, a flake size of ~13.5 μm was observed (Fig. S1a). A large flake size could facilitate charge transfer during electrochromic reactions. Due to the surface groups (‒OH and =O) induced during the chemical etching process, the Ti_3_C_2_ solution showed a high negative zeta potential and thus exhibited good colloidal stability (see Fig. S1b).

**Fig. 1:**
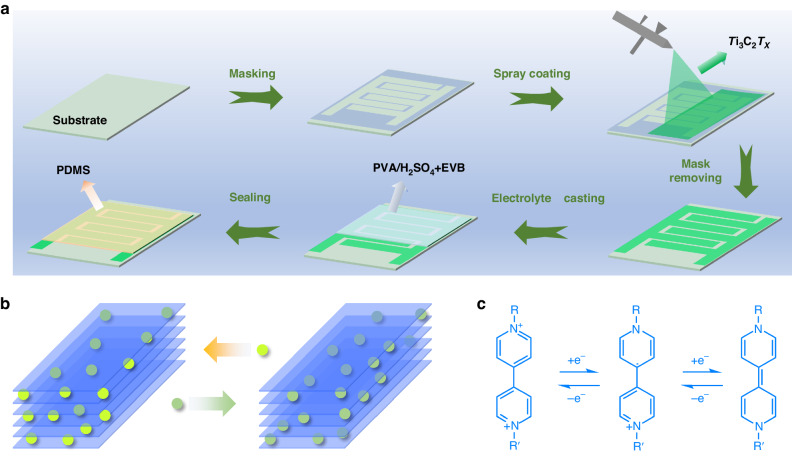
**Schematic illustration of the in-plane device**. Assembly process (**a**), ion transfer process (**b**), and redox reaction of the EVB molecules (**c**)

The fabrication process of the planar device is schematically illustrated in Fig. 1a. Interdigitated Ti_3_C_2_ MXene thin films were deposited onto transparent substrates, such as rigid glass slides or flexible PET films, by a mask-assisted spray coating approach. Due to its in-plane electrode architecture, the device has the potential to be miniaturized and integrated into energy storage units in portable electronics. As shown in Fig. 1b, the interspaces between the fingers allow the elimination of the separators, which further increases the accessibility of the electrolyte ions to the edges of the electrodes. In addition, the ions move parallel to, instead of perpendicular to, the 2D Ti_3_C_2_ flakes; this movement decreases the ion transport resistance of the film electrodes. As a result, these devices show promising high-frequency responses and high-power performance. Moreover, compared with traditional stacked sandwich devices, microsupercapacitors with a planar architecture have better flexibility and mechanical stability.

Next, EVB was mixed with the PVA/H_2_SO_4_ gel electrolyte to prepare the electrochromic electrolyte, which was drop-cast onto the surface of the in-plane device and solidified overnight. Finally, an all-solid-state EMS was constructed after sealing with PDMS. Notably, EVB is a widely used viologen derivative for electrochromic devices, showing significant color change between its colorless and deep purple states; this is attributed to its fast and highly reversible two-step redox reaction (see Fig. 1c)^32^. As a result, the obtained devices show fast and reversible color changes during the charge/discharge process, accompanied by high optical contrast and excellent CE.

**Fig. 2:**
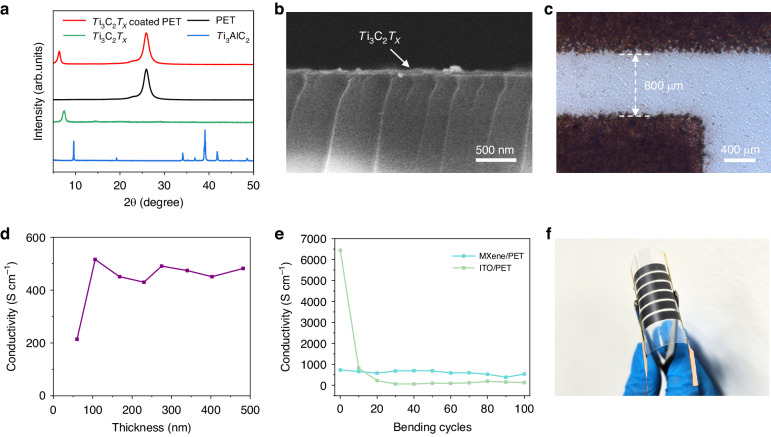
**Performance of the Ti**_**3**_**C**_**2**_**/PET film**. **a** XRD patterns of the Ti_3_AlC_2_ MAX, Ti_3_C_2_, PET, and Ti_3_C_2_-coated PET films. **b** Cross-sectional SEM image of the spray-coated Ti_3_C_2_ film. **c** Top-view optical microscope image of the in-plane device. **d** Relationship between the conductivity and thickness of the Ti_3_C_2_ film. **e** Conductivity change and **f** digital image of the Ti_3_C_2_/PET film during the bending test

The crystal compositions of the Ti_3_AlC_2_ MAX, Ti_3_C_2_ film, and Ti_3_C_2_-coated PET were initially investigated by X-ray diffraction (XRD). The (002) peak of Ti_3_AlC_2_ at 9.6° shifted to 6.3° after the chemical etching process, and the other peaks disappeared; these results indicated the successful synthesis of Ti_3_C_2_ MXene (see Fig. 2a). No other peaks were observed in Ti_3_C_2_ coated PET, confirming the high purity of the spray-coated Ti_3_C_2_ film. Notably, the (002) peak of the spray-coated Ti_3_C_2_ film showed a slight shift toward a lower angle compared with that of the filtered film; these results indicated larger interlayer spacing between the sprayed MXene flakes, which could therefore induce a faster ionic transport rate during the electrochemical process.

To ensure a short ion diffusion pathway, a series of EMSs were designed with a constant gap of 800 μm and varying finger width. A series of fingers with widths of 2000, 3000, 4000, and 5000 μm were fabricated to optimize the performance of the device; these fingers were named EMS-2, EMS-3, EMS-4, and EMS-5, respectively (see Fig. S2). As shown in the cross-sectional SEM image of Fig. 2b, the compact stacked structure of the spray-coated Ti_3_C_2_ film had a thickness of ~60 nm. In addition, the top-view optical microscope image of the fingers exhibited regular edges (Fig. 2c), and these edges were attributed to the regular shape of the mask and beneficial for ion transport.

The optical transmittance and electronic conductivity of the Ti_3_C_2_ electrodes are important parameters for electrochromic and energy storage devices. First, the transmittance of the Ti_3_C_2_ films with different thicknesses in the visible region was measured, and a broad peak was observed in the visible region (Fig. S3). The 60 nm film showed a transmittance of ~70% at 550 nm, which decreased with increasing thickness. In addition, the relationship between the electronic conductivity and thickness was revealed for further applications, as shown in Fig. 2d. The highest electrical conductivity of 510 S cm^−1^ was obtained at a thickness of 106 nm, which remained constant with further increases in thickness.

Notably, the as-prepared Ti_3_C_2_/PET films showed excellent flexibility (see Fig. 2e, f). A bending test was performed to investigate the mechanical stability of the Ti_3_C_2_/PET film at a bending radius of 5 mm, and its mechanical stability was much greater than those of widely used commercial ITO/PET films. No obvious change was observed in the conductivity of the Ti_3_C_2_/PET film after 100 bending and releasing cycles, while the conductivity of the ITO/PET film sharply decreased by 2 orders of magnitude during the first 20 bending cycles. These results confirmed the excellent mechanical stability of the Ti_3_C_2_/PET film, and this stability was attributed to the 2D structure of the Ti_3_C_2_ flakes, which exhibited strong van der Waals forces with the adjacent layers.

**Fig. 3:**
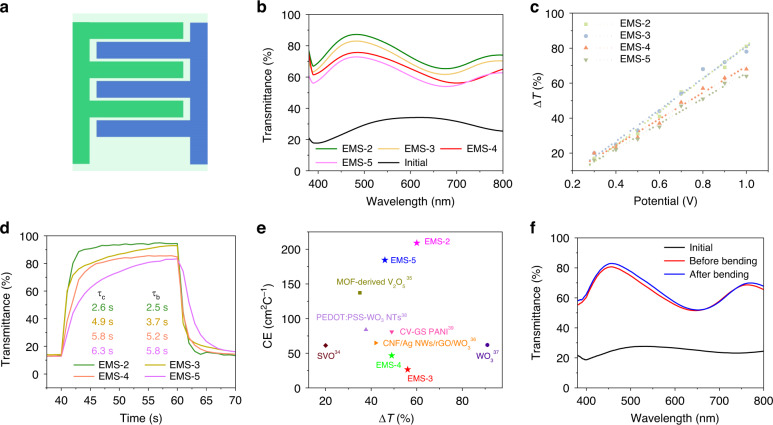
**Electrochromic performance of the in-plane EMS**. **a** Schematic illustration and **b** UV‒vis spectra of the EMSs at voltages of 0 V and 1 V. **c** Optical modulation range of various EMSs at different voltages. **d** Comparison of the coloration/blenching times of the EMSs. **e** Relationships between coloration efficiency and Δ*T* for the EMSs and previously reported electrochromic supercapacitors^31–36^. SVO: sodium ion-stabilized vanadium oxide, MOF: metal–organic framework, CNF: cellulose‐nanofiber, Ag NWs: Ag‐nanowires, rGO: reduced graphene oxide, WO_3_ NTs: WO_3_ nanotube, CV: cyclic voltammetry, GS: galvanostatic, PANI: polyaniline. **f** Electrochromic switching behavior of EMS-2 after 100 bending cycles

Due to the significant pseudocapacitive reactions between the proton and surface terminations of MXenes, Ti_3_C_2_ has been extensively studied as a high-performance supercapacitor electrode in H_2_SO_4_-based aqueous electrolytes. Thus, to ensure high charge storage of the device, 1 M PVA/H_2_SO_4_ with EVB was selected as the solid-state electrolyte for the EMSs. The electrochromic performance of the EMSs was initially measured and compared by in situ UV‒vis spectroscopy (see Fig. S4). Due to the excellent electrochromic performance of the EVB molecule, the negative electrodes of the EMSs showed a significant color change from deep green to blue with increasing applied voltage (Fig. 3a). As shown in Fig. S5, the transmittance of EMS-2 at 550 nm increased from ~20% to ~80% as the voltage increased to 1 V. Additionally, the peaks of the UV‒vis spectra showed a significant blueshift, corresponding to an evident color change. Notably, the electrochemical performance of the in-plane device was highly correlated to the width of the electrodes, which could influence the amount of charge transfer during the electrochemical process^33^. Thus, the maximum optical modulation ranges (Δ*T* = *T*_b_ − *T*_c_, where *T*_b_ and *T*_c_ are the transmittances of the bleach and colored states, respectively) of the four devices were significantly different (see Fig. 3b). Under the driving voltage of 1 V, EMS-2, EMS-3, EMS-4, and EMS-5 exhibited Δ*T* values of 60%, 56%, 49%, and 46%, respectively. The Δ*T* gradually decreased with increasing width of the electrode, and this result is likely attributed to the higher charge density of the electrode with narrower fingers, inducing a deeper redox reaction degree of the EVB molecule in the electrolyte.

The Δ*T* of the EMSs under different trigger voltages were further quantified and are shown in Fig. 3c; here, the scatter plots could be fitted to a straight line for each device, and the direct proportionality was observed between Δ*T* and the applied voltage (see Fig. S6). This good linearity was ascribed to the fast redox reactions of the EVB molecules and the superior charge transfer ability of the Ti_3_C_2_ film. The coloration time (*τ*_c_) and bleaching time (*τ*_b_) are other important characteristics for evaluating electrochromic devices and are denoted as the time needed to achieve 90% of the total optical modulation range. The temporal response of the transmittance of the EMS devices at 483 nm was recorded under the periodic applied pulse voltages of 0 and 1 V (Fig. S7). As shown in Fig. 3d, four EMS devices also exhibited varying coloration/bleaching times, which increased with increasing electrode width (2.6/2.5 s, 4.9/3.7 s, 5.8/5.2 s, and 6.3/5.8 s for EMS-2, EMS-3, EMS-4, and EMS-5, respectively). The rapid color change of the EMS-2 device could be attributed to the fast charge transfer on the narrow electrode fingers. However, as the electrode width increased, the ionic transport pathway became longer, resulting in relatively slow charge transfer.

In addition, the CE is another key performance indicator of electrochromic devices; CE is defined as follows: CE = ΔOD/*Q*, where ΔOD represents the change in the optical density (lg(*T*_b_/*T*_c_)) and *Q* represents the pumped charge density. The EMS-2, EMS-3, EMS-4, and EMS-5 devices had CEs as high as 209.0, 26.5, 46.6, and 184.4 cm^2^ C^−1^, respectively; these results indicated that a small number of inserted charges could lead to a large optical modulation range. Notably, the CEs were superior to those of previously reported electrochromic devices (see Fig. 3e)^34–39^. This excellent coloration ability was caused by the fast charge transfer of the EVB molecule and the highly conductive Ti_3_C_2_ flakes, facilitating the utilization of the injected charges.

The mechanical stability is significant for the practical application of flexible devices. As shown in Fig. 3f, after 100 bending cycles, the optical switching performance of EMS-2 showed only a 5% decrease from the original value, indicating its significant mechanical stability and promising potential for flexible electronics. This high mechanical stability of the 2D MXene films was attributed to the simplified interdigitated planar structure, which could buffer the stress within the different layers of the device.

**Fig. 4:**
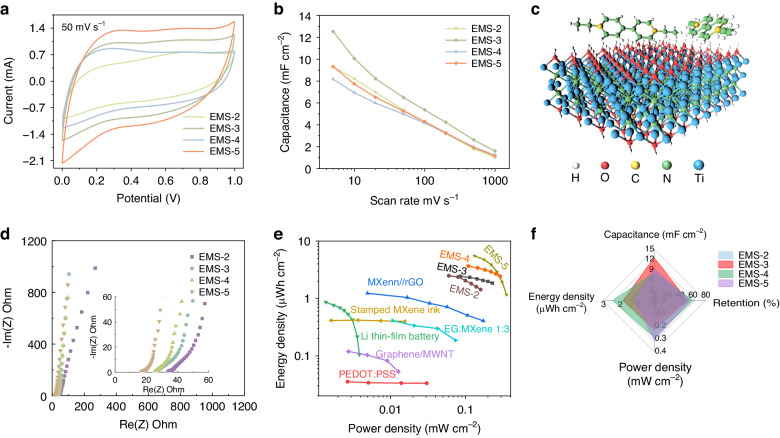
**Energy storage performance of the EMSs**. **a** CV curves of the EMSs at a scan rate of 50 mV s^−1^. **b** Areal capacitance comparison of the devices at different scanning rates. **c** Schematic illustration of the binding between the surface terminations of the MXene flakes and EVB molecules. **d** Nyquist plots of the EMSs. **e** Ragone plot of the four EMSs compared with the reported microsupercapacitors. **f** Comprehensive evaluation diagram of the four EMSs

Cyclic voltammetry (CV) was further conducted to evaluate the energy storage behavior of the EMSs (see Fig. 4a and Fig. S8). All four devices exhibited a stable working voltage window of 1 V, with evident redox peaks. The areal-specific capacitance values of the EMSs under various scan rates were also calculated, and a comparison is shown in Fig. 4b. The highest capacitance of 12.5 mF cm^−2^ was achieved by EMS-3 at 5 mV s^−1^. Due to the high conductivity of the Ti_3_C_2_ film and the fast redox reactions, more than 50% of the capacitance could be retained for all four devices as the scan rate increased to 100 mV s^−1^. EMS-2, EMS-3, EMS-4, and EMS-5 exhibited specific capacitances of 9.3, 12.5, 8.2, and 9.3 mF cm^−2^ at 5 mV s^−1^, respectively. Additionally, the four EMSs showed different high-rate retention abilities, which were caused by the balance between the electronic transport and ionic transport since wider fingers usually had better electronic transport abilities with longer ionic transport pathways.

Notably, Ti_3_C_2_ MXene flakes are sensitive to the applied voltage and should be oxidized under a positive voltage. As a result, Ti_3_C_2_-based electrodes are generally used as negative electrodes, and the voltage window of Ti_3_C_2_-based symmetric aqueous devices is limited to 0.6 V^19^. As shown in Fig. S9a, the voltage window of the device using 1 M PVA/H_2_SO_4_ (without EVB) as the electrolyte was limited to 0.8 V, which showed rapid decay of the capacitance in the first several cycles (see Fig. S9b). The results indicated that the addition of EVB could enhance the stability of the Ti_3_C_2_ film under a positive voltage, and a similar phenomenon was also reported for polyaniline in our previous work^40^. The binding of the Ti_3_C_2_ flakes and EVB molecules could induce a shift in the Fermi level, which resulted in a large resistance to electron loss and a greater ability to withstand anodic oxidation (Fig. 4c). In addition, the CV curves of the EMSs exhibited evident pseudocapacitive behavior instead of the ideal rectangular shape of the device without EVB molecules, further demonstrating that EVB is a kind of redox-active electrolyte that improves the charge storage ability.

Moreover, electrochemical impedance spectroscopy (EIS) analysis was conducted, and the results are shown in Fig. 4d. All Nyquist plots of the four devices showed nearly vertical slopes in the low-frequency regime, indicating close-to-ideal capacitive behavior. The equivalent series resistance in the high-frequency region decreased with increasing finger width, which was 34.4, 26.3, 25.6, and 17.4 Ω for EMS-2, EMS-3, EMS-4, and EMS-5, respectively; these results indicated that a wider finger showed better electronic transport ability.

The galvanostatic charge‒discharge (GCD) profiles were tested at various current densities and exhibited good agreement with the CV curves and good symmetric characteristics; thus, these devices had high coulombic efficiency (Fig. S10). The Ragone plot in Fig. 4e shows the energy density and power density of the EMSs. The four devices (EMS-2, EMS-3, EMS-4, and EMS-5) showed high energy densities (power densities) of 2.48 μWh cm^−2^ (0.16 mW cm^−2^), 2.42 μWh cm^−2^ (0.23 mW cm^−2^), 3.66 μWh cm^−2^ (0.29 mW cm^−2^), and 5.53 μWh cm^−2^ (0.35 mW cm^−2^), respectively. The improved energy and power densities of the EMSs were caused by the wide working voltage window of the devices, and these values were superior to those of previously reported microsupercapacitors^41–45^. In addition, the typical energy storage performances of the four EMSs were further compared in the radar image (Fig. 4f).

To further demonstrate the applicability of the coplanar device in different electrolytes, we assembled EMS-3 devices using a 1 M LiCl electrolyte containing EVB, as shown in Fig. S11. The device exhibited a stable voltage window of 1.2 V, with a capacitance of 11.3 mF cm^−2^ at a scan rate of 5 mV s^−1^. When the scan rate was increased to 1000 mV s^−1^, the capacitance decreased to only 0.6 mF cm^−2^. This result was consistent with our previous findings, indicating that metal salt electrolytes produced a wider voltage window than acidic electrolytes with a lower capacitance and rate capability^46^. Regarding the color change performance, at a voltage of 1.2 V, the maximum optical modulation range at 550 nm was 64%; this value was also lower than that observed with acidic electrolytes.

**Fig. 5:**
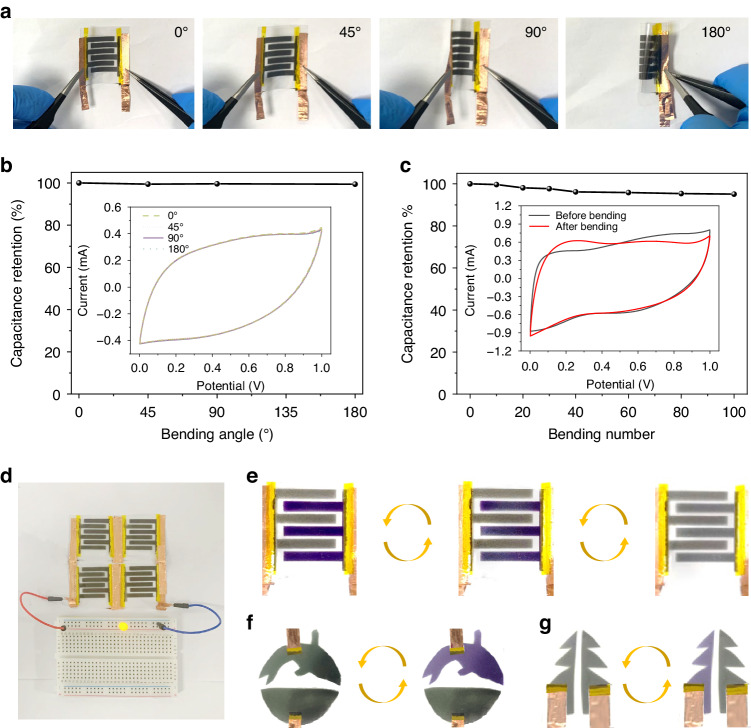
**Application of the as-fabricated EMS**. **a** Digital images of EMS at 0–180° bending angles. **b** Capacitance retention at different angles (inset: CV curves at a scan rate of 50 mV s^−1^). **c** Capacitance retention of EMS after bending 100 times at 180° (inset: CV curve before and after bending 100 times at a scan rate of 50 mV s^−1^). **d** Digital image of an LED illuminated by four EMS-3 devices in series and parallel. **e** EMS-3 under different charging states (fully charged, half charged, and empty from left to right), and **f**, **g** On-surface patterned devices

Flexibility and mechanical stability are critical for practical applications of flexible or wearable electronics. The flexibility and mechanical stability were evaluated for EMS-3, which exhibited the best overall performance (see Fig. 4f). As shown in Fig. 5a, the device could be bent from 0° to 180° with stable electrochemical performance (Fig. 5b). The CV curves showed negligible changes under different bending states, demonstrating the outstanding flexibility and stability of the device. In addition, the device could be bent many times during application, showing its excellent mechanical stability of EMS under bending conditions. Figure 5c illustrates the capacitance retention of EMS-3 during repeated 180° bending cycles. Even after 100 bending cycles, 95% of the initial capacitance remained, indicating excellent stability under extreme usage conditions. Moreover, EMS-3 devices could be connected in series or parallel to power light-emitting diodes (LEDs) (see Fig. 5d); this result indicated the significant potential of EMSs for powering devices in various fields through series and parallel connections to alter the operating voltage. Figure 5e shows the performance of the device in the three energy storage states of full, half-full, and empty with real-time determination facilitated by the color depth, highlighting the device’s convenience in usage. Figure 5f, g shows different patterned devices of surface-mounted EMSs, indicating patternability based on the different needs or occasions for personalized customization. This versatility enables a wide-range applications in areas such as camouflage, anticounterfeiting, and displays.

## Conclusion

In summary, a flexible planar interdigitated EMS based on symmetric Ti_3_C_2_ MXene finger electrodes was fabricated using a mask-assisted spray coating technique. The device exhibited high areal capacitance of 12.5 mF cm^−2^, with good rate capability, in a 1 M PVA/H_2_SO_4_ gel electrolyte containing EVB molecules. The addition of EVB not only enhanced the capacitance of the device but also facilitated the anodic oxidation resistance of the Ti_3_C_2_ flakes to increase the working voltage window. In addition, the devices showed highly reversible and rapid electrochromic performance (from deep green to blue for the negative electrode), a fast response time (2.6 s/2.5 s), high optical contrast (60%), and exceptional coloration efficiency (209 cm^2^ C^−1^). The color change accompanying the energy storage process indicated the charging state of the device. Notably, the device demonstrated significant mechanical stability, with only negligible decreases in both the energy storage and electrochromic performances even after hundreds of bending cycles. The flexible dual-function in-plane device has potential for application in flexible and wearable electronics, particularly in the fields of camouflage, anticounterfeiting, and displays.

## Methods

### Materials

All chemical reagents were used as received without further purification. Ti_3_AlC_2_ powder (98%, 400 mesh) was purchased from Jilin 11 Technology Co., and hydrochloric acid (HCl, 36%~38%) and sulfuric acid (H_2_SO_4_, 95%~98%) were obtained from China National Pharmaceutical Group Chemical Reagent Co., and lithium fluoride (LiF, ≥98%) was obtained from Jinan Maina Technology Co., Ltd. Ethyl violet dibromide was purchased from Shanghai Aladdin Bio-Chem Technology Co., Ltd. Polyvinyl alcohol (PVA, *M*_w_ = 98,000) was acquired from Shanghai Macklin Bio-Chem Technology Co., Ltd.

### Preparation of Ti_3_C_2_ MXene

Ti_3_C_2_ MXene was synthesized through the etching of Ti_3_AlC_2_ in a solution created by introducing LiF salt into an HCl solution. The etching solution was prepared by mixing 1 g of LiF with 15 mL of 12 M HCl and stirring for 5 min. Subsequently, 1 g of Ti_3_AlC_2_ powder was slowly introduced into the aforementioned etchant over a few min and continuously stirred at 35 °C for 24 h. The resulting acidic suspension of Ti_3_C_2_T_*x*_ was rinsed with DI water via centrifugation at 3500 rpm for 5 min in each cycle, and the supernatant was decanted. Upon reaching a pH of ~6, a stable, dark supernatant of Ti_3_C_2_T_*x*_ was observed and collected following 30 min of centrifugation at 3500 rpm. The ink-like supernatant, with a concentration of 2 mg mL^−1^, was collected and subsequently utilized for spray coating.

### Fabrication of the MXene-based devices

#### Substrate pretreatment

The PET substrate was first subjected to a cleaning process using DI water and ethanol to eliminate contaminants. It was subsequently dried using nitrogen (N_2_) gas. Finally, the substrate underwent an additional surface treatment procedure utilizing a plasma cleaning machine.

#### Preparation of the gel electrolyte

Initially, 1 g of PVA was dissolved in 10 mL of DI water, heated to 85 °C, and stirred continuously for 3 h until a clear, transparent gel was formed. Next, 1 g of concentrated H_2_SO_4_ was added to the 10 wt% PVA gel and stirred for 30 min. Subsequently, 50 mg of ethyl violet was introduced into the mixture and stirred continuously for 1 h; this resulted in the creation of a PVA/H_2_SO_4_/ethyl violet gel electrolyte with a concentration of 5 mg mL^−1^.

#### Assembly of MXene-based devices

AutoCAD technical drawing software was utilized to design the masks, and these masks were then employed in the fabrication of the MXene-based interdigital film electrodes. The custom interdigital mask consisted of three finger-like structures on each side, with a gap width of 800 μm and finger widths ranging from 2000 to 5000 μm. The template was placed and secured onto a pretreated PET substrate. A low-cost spray gun was used to spray the MXene, and after each deposition, a hairdryer was used to dry the deposited layer. The resistance of the film electrode was periodically measured with a multimeter until it reached ~200 Ω. Subsequently, the obtained all-MXene interdigital electrode was placed in a vacuum oven at 60 °C overnight to remove any residual moisture. Then, the PVA/H_2_SO_4_/ethyl violet gel electrolyte was carefully drop-cast uniformly onto the surface of the interdigital patterns. The gel was left undisturbed for 30 min for semi-solidification. Prior to this, copper tape was used to connect to the electrode, and a thin layer of conductive silver paste was applied to improve their ohmic contact. Afterward, Kapton tape was used to cover the conductive silver paste; this tape prevented the paste from reacting with the electrolyte and protecting the paste from oxidation. Finally, an additional layer of PDMS was applied to encapsulate the gel electrolyte, and this layer served as a passivation layer to further enhance the stability.

### Characterization

SEM images were obtained by field emission scanning electron microscopy (Hitachi S-4800). XRD patterns were obtained with an X-ray diffractometer (Bruker AXS D8 Advance) using Cu Kα radiation (*λ* = 1.5406 Å) over the range of 2*θ* = 5.0~50.0°. The thin-film sheet resistance was tested with a Desili Electric digital multimeter (DEM21). Raman spectra were collected with a laser microconfocal Raman spectrometer (Renishaw inVia). The electrochromic performance was assessed using a combination of an electrochemical workstation (CHI660E) and a marine optical spectrometer (HR-2VN400-25). The electrochemical measurements, including CV, GCD, and EIS, were carried out on an electrochemical workstation.

### Supplementary information

Supplementary file
