# Supplementary material for: MXene-based all-solid flexible electrochromic microsupercapacitor
Source: Microsyst Nanoeng. 2024 Jun 25;10:89. doi: 10.1038/s41378-024-00720-6 (PMC11196698; doi:10.1038/s41378-024-00720-6)
Supplement: Supplementary file 1 — Supplementary file [file 41378_2024_720_MOESM1_ESM.docx]

Supporting Information

**MXene-based all-solid flexible electrochromic microsupercapacitor**

Shanlu Guo^1,#^, Ruihe Zhu^1,#^, Jingwei Chen^2^, Weilin Liu^1^, Yuxiang Zhang^1^, Jianmin Li^1,*^, Haizeng Li^3,4,5,*^

^1^ College of Electronic and Optical Engineering & College of Flexible Electronics (Future Technology), Nanjing University of Posts and Telecommunications, Nanjing 210023, China

^2^ School of Materials Science and Engineering, Ocean University of China, Qingdao, 266100 China

^3^ Optics and Thermal Radiation Research Center, Institute of Frontier and Interdisciplinary Science, Shandong University, Qingdao, 266237, P. R. China

^4^ Shenzhen Research Institute of Shandong University, Shenzhen, 518000, China

^5^ State Key Laboratory of Featured Metal Materials and Life-Cycle Safety for Composite Structures, Guangxi University, Nanning, 530004, China


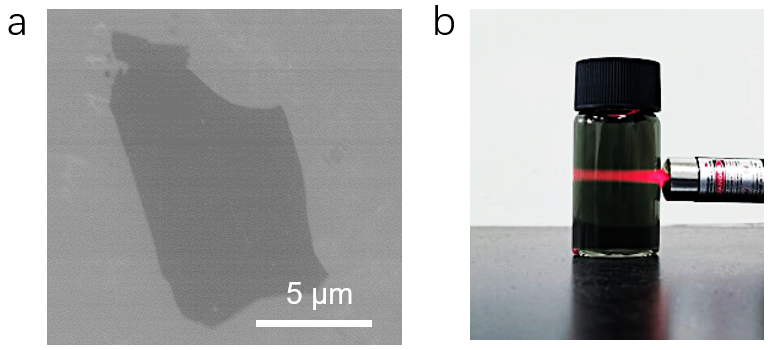


**Fig. S1**. (a) SEM image of the single-layer Ti_3_C_2_ flake. (b) Tyndall effect image of MXene dispersion.


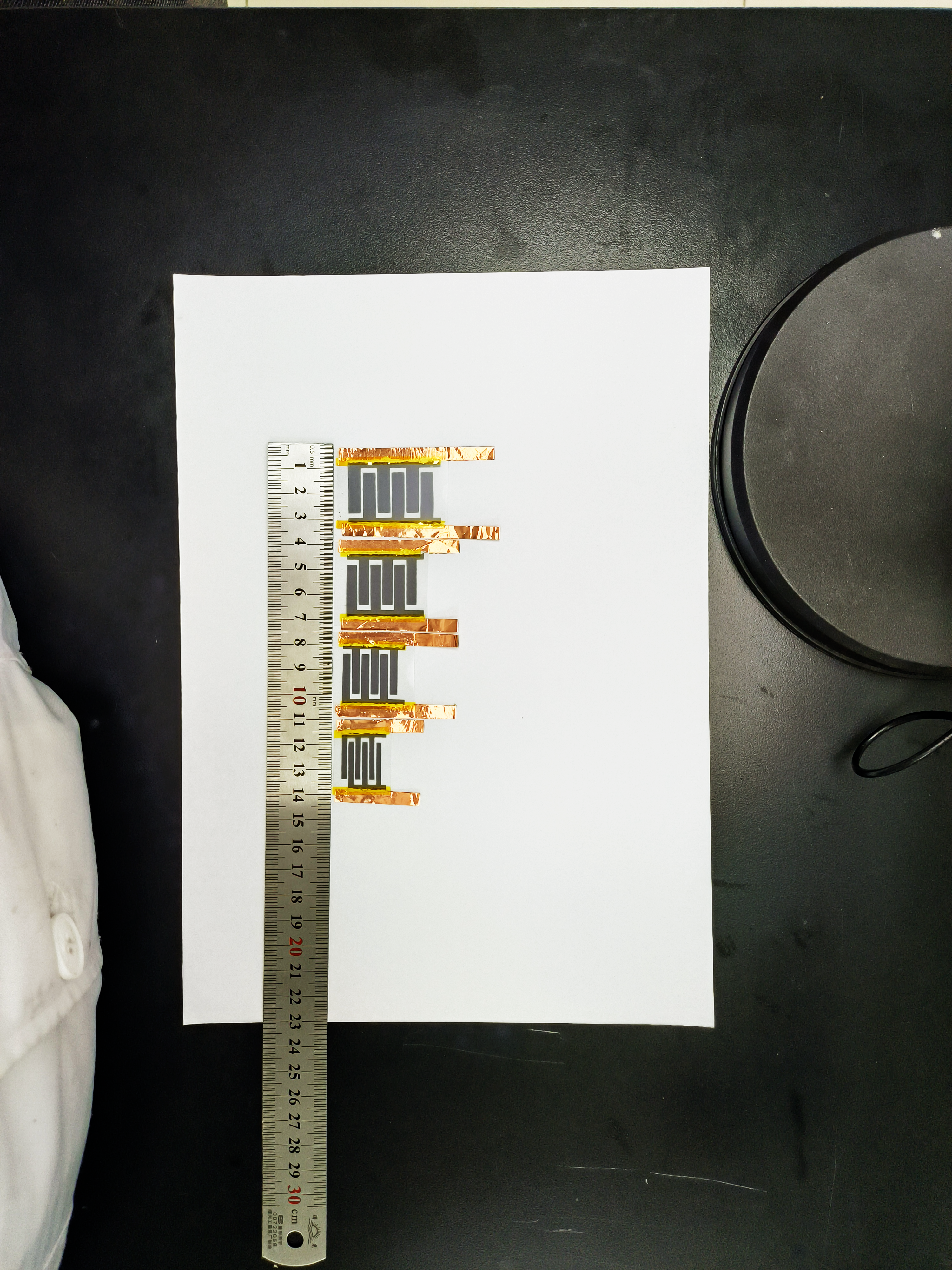


**Fig. S2**. The digital images of EMS-2, EMS-3, EMS-4, and EMS-5 from right to left.


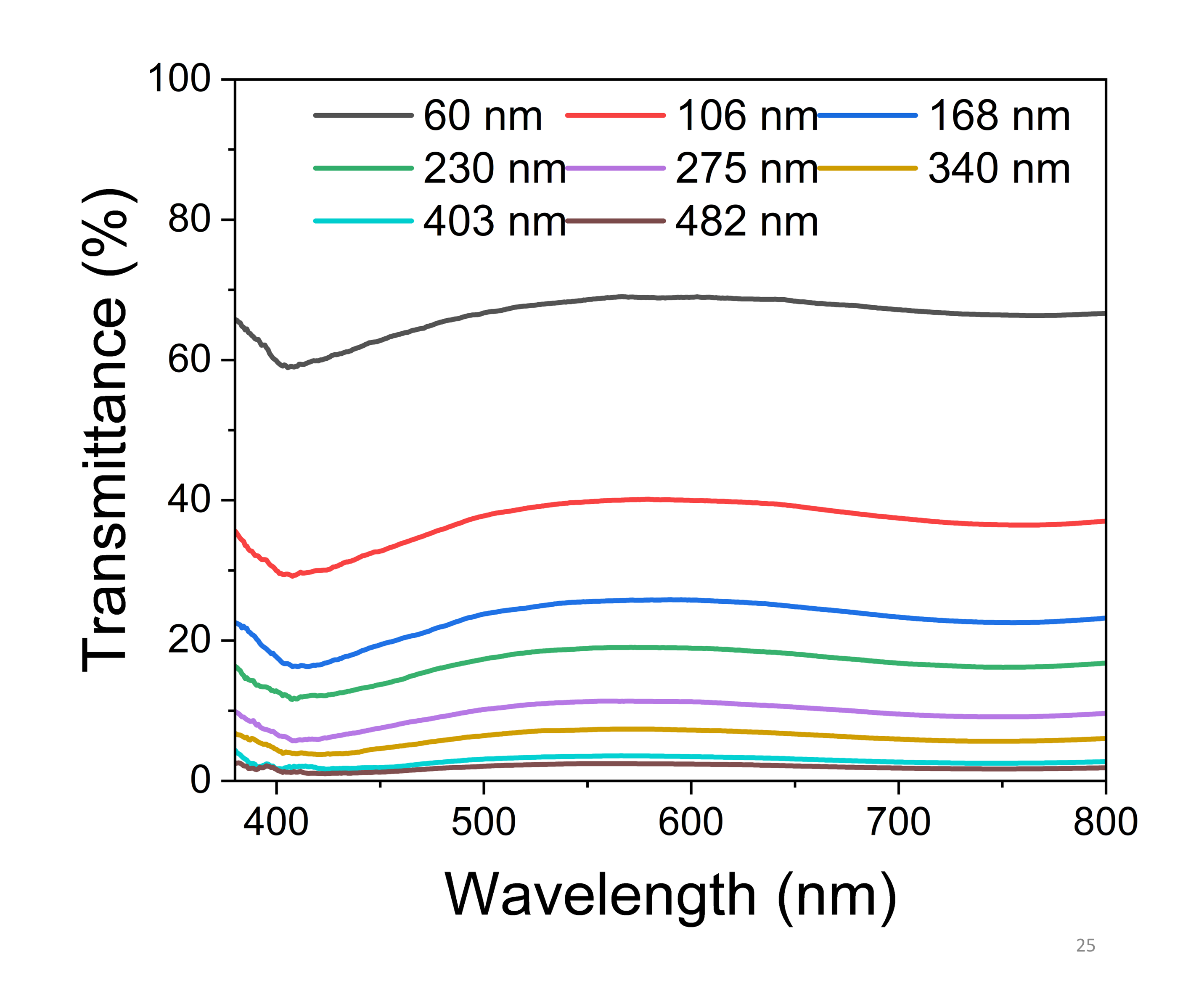


**Fig. S3**. The UV-Vis spectra of spray-coated MXene films with different thicknesses.


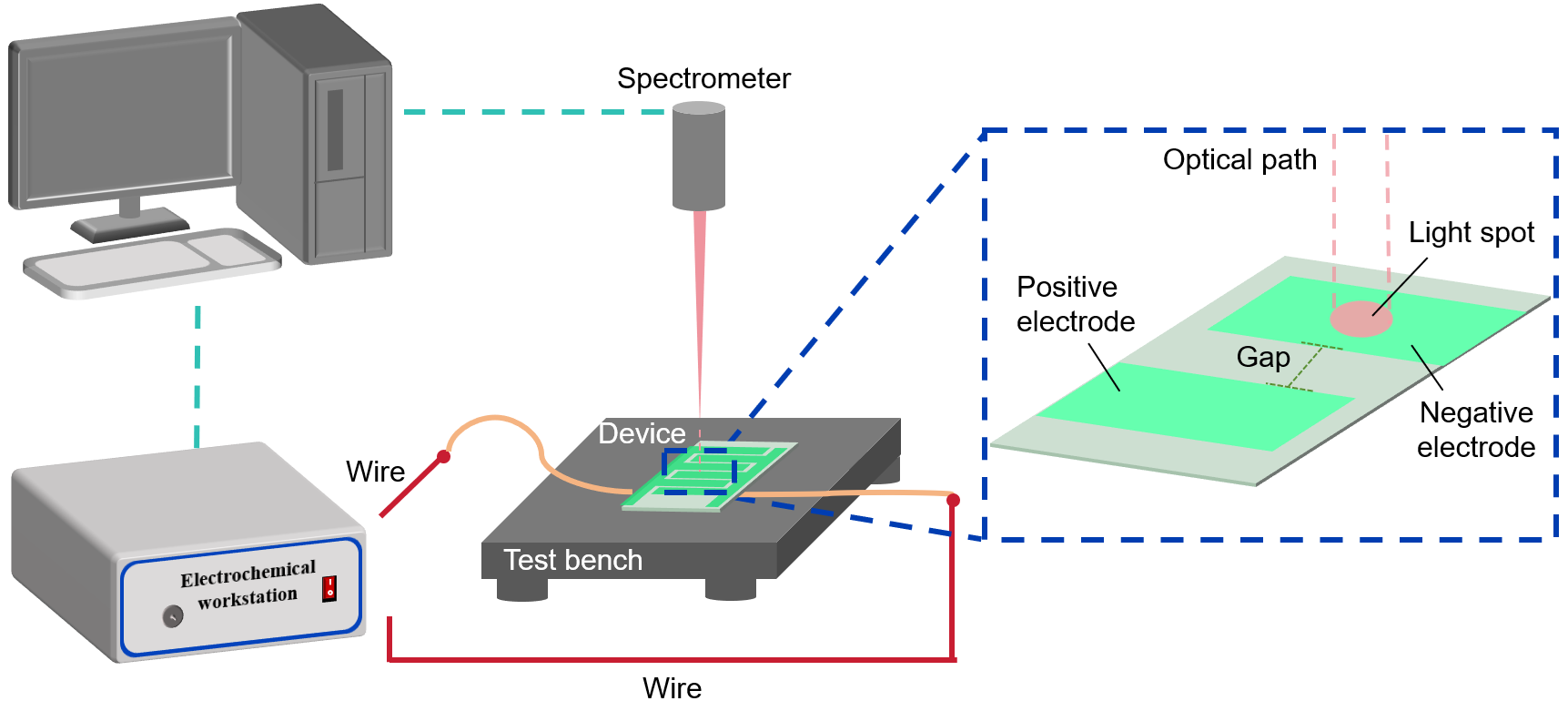


**Fig. S4**. The schematic illustration of the in-situ UV-Vis spectrum test.


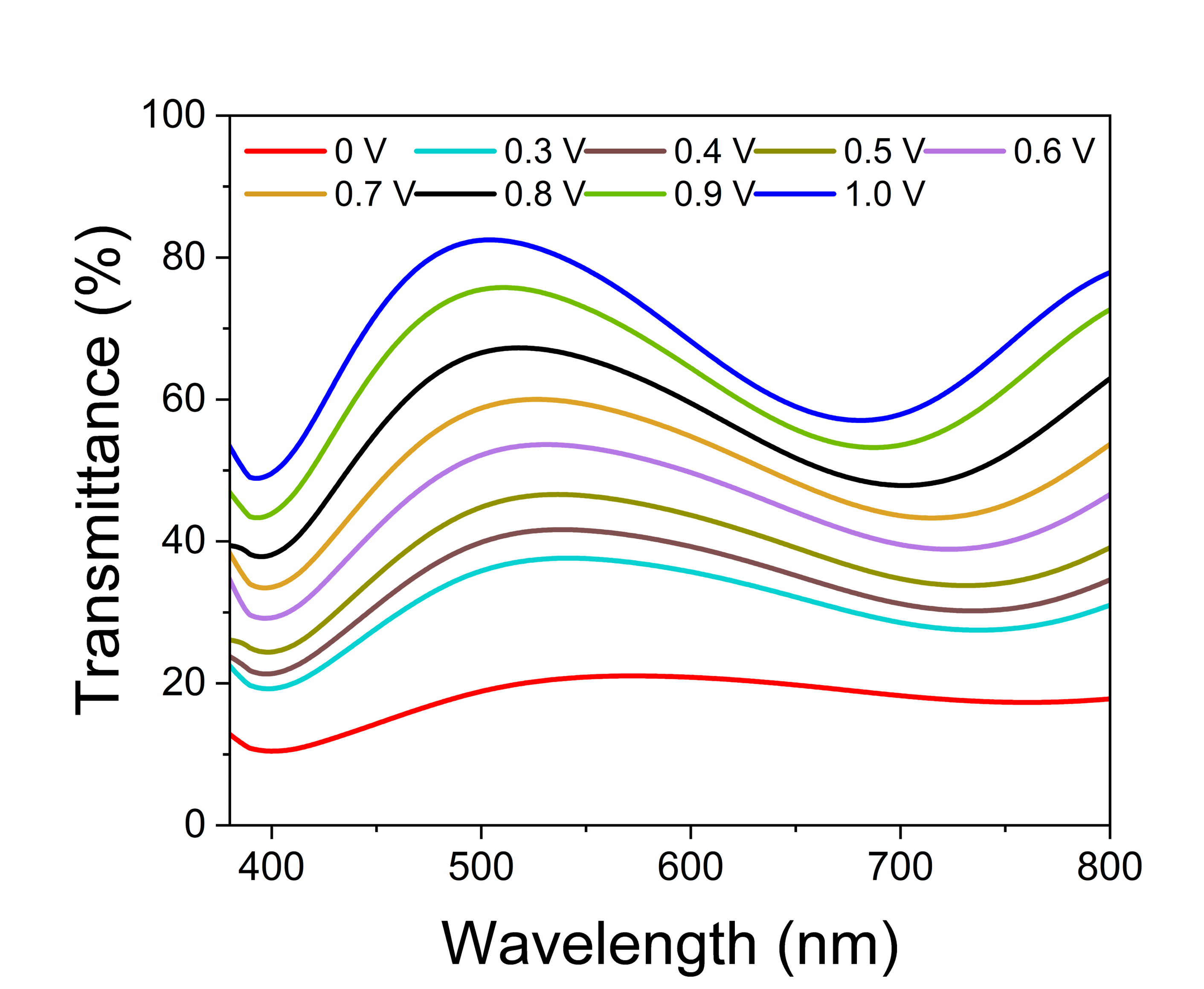


**Fig. S5**. The initial UV-Vis spectrum of the device under different voltages.


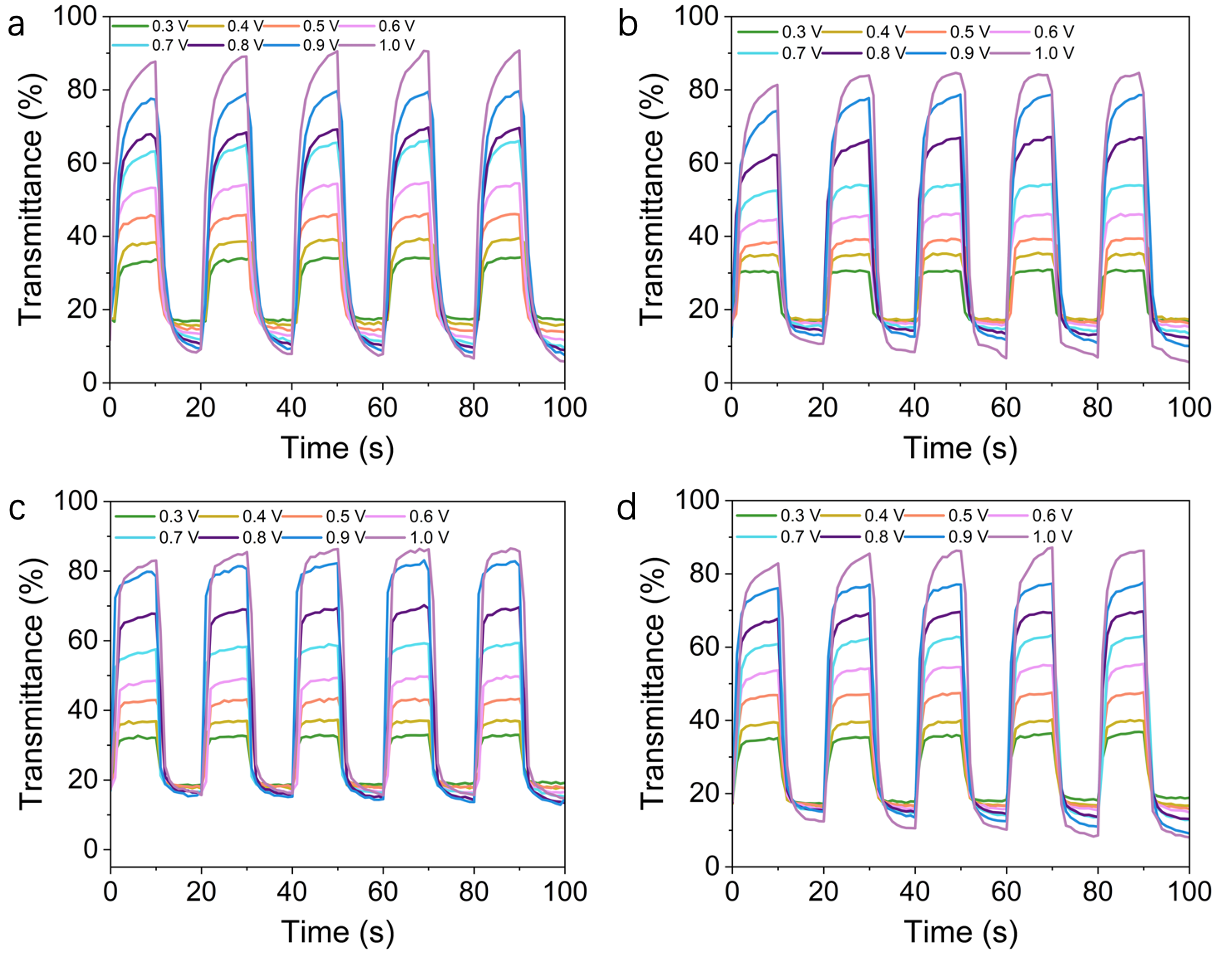


**Fig. S6**. Transmittance change of (a) EMS-2, (b) EMS-3, (c) EMS-4, and (d) EMS-5 at 550 nm, under different voltages.


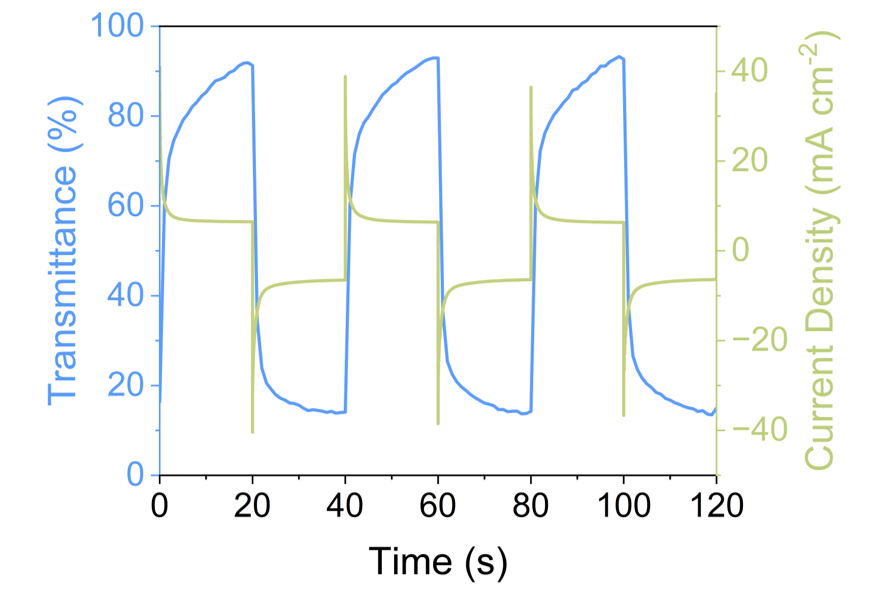


**Fig. S7**. Time response of the transmittance spectrum under pulsed voltage.


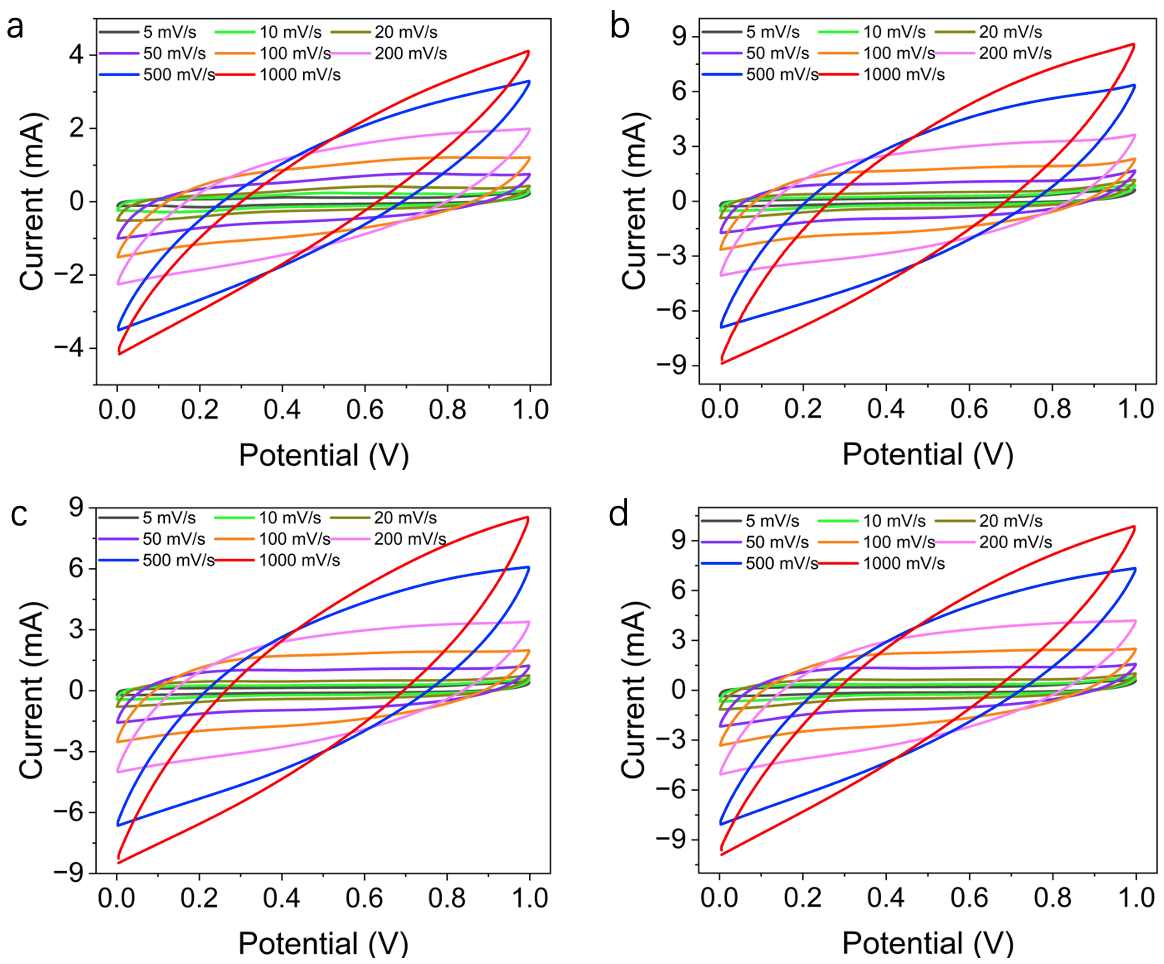


**Fig. S8**. The CV curves of (a) EMS-2, (b) EMS-3, (c) EMS-4, and (d) EMS-5, under various scan rates.


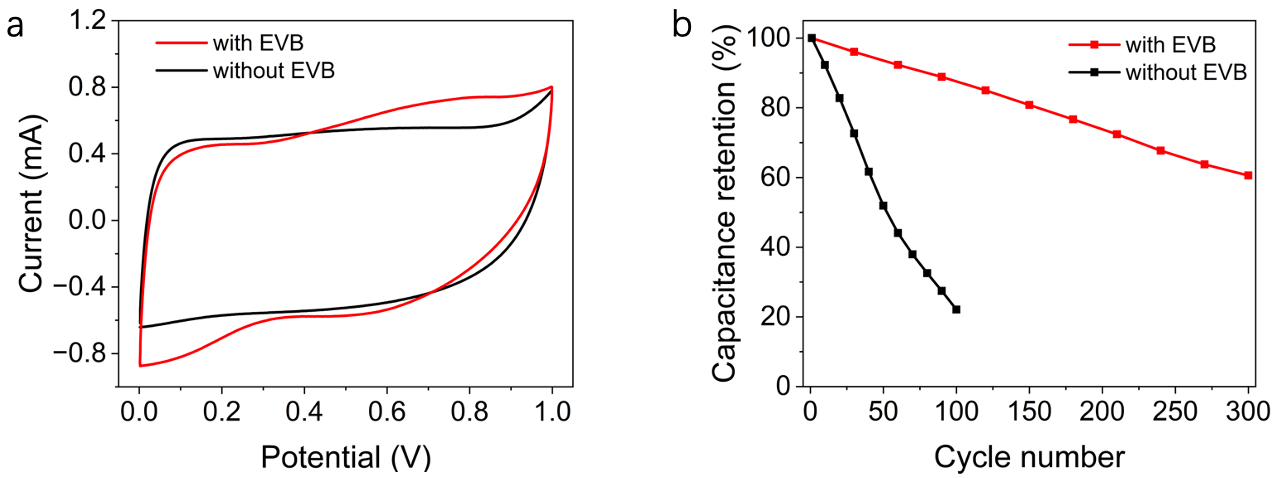


**Fig. S9**. (a) CV curves and (b) cycle stability of the devices using the electrolyte with and without EVB.


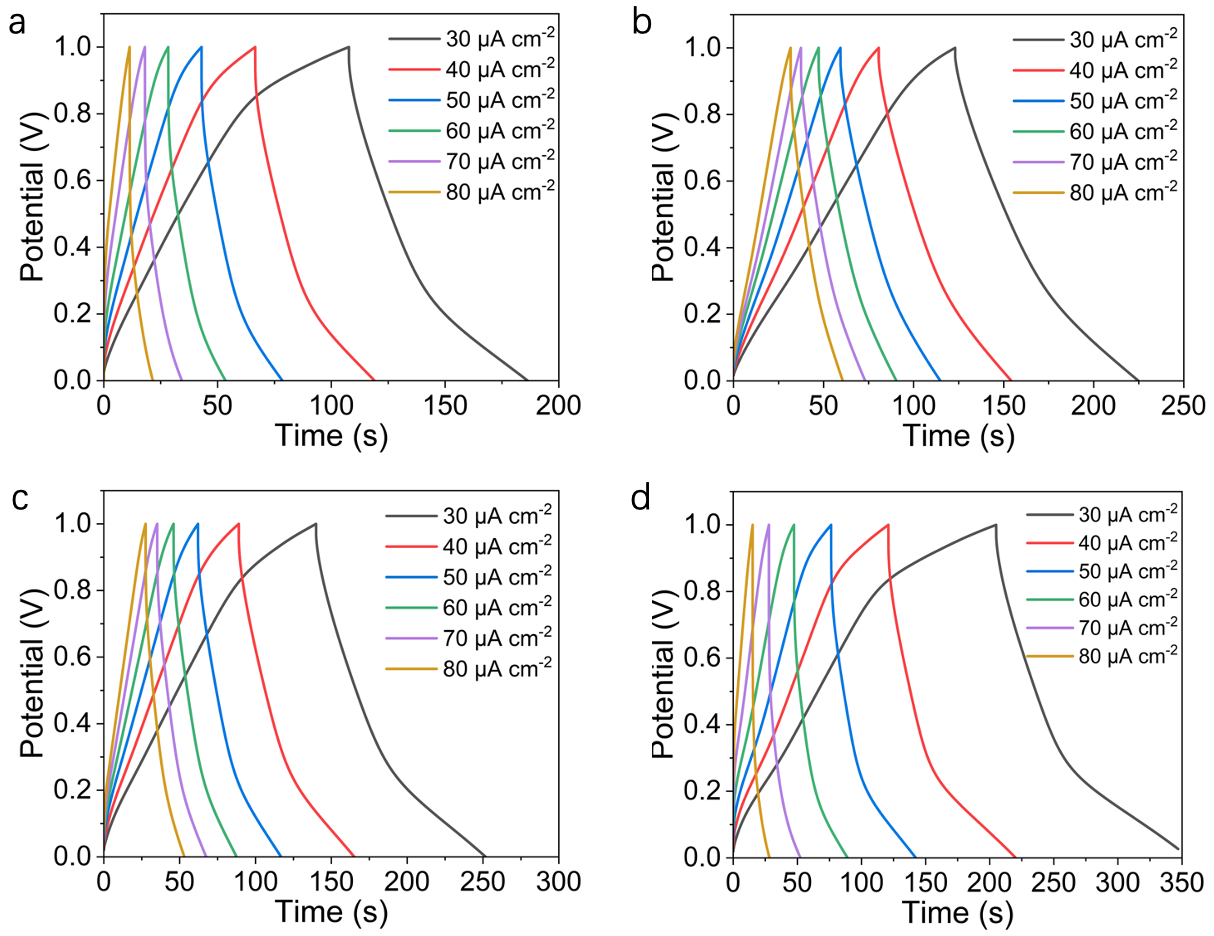


**Fig. S10**. GCD curves of (a) EMS-2, (b) EMS-3, (c) EMS-4, and (d) EMS-5, under various current density.


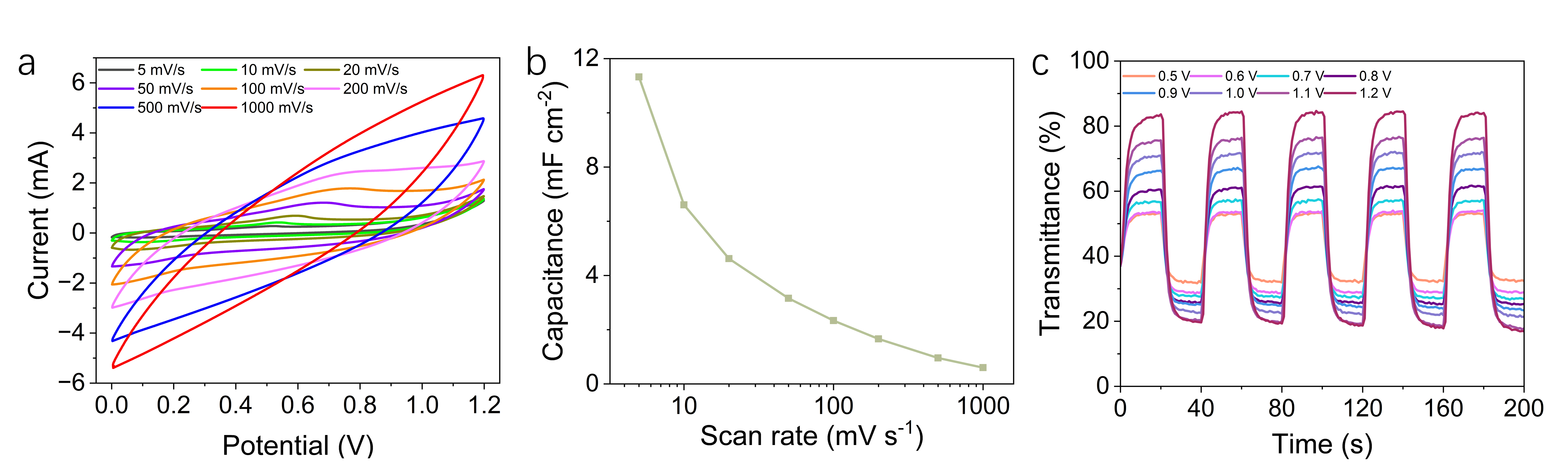


**Fig. S11**. (a) CV curves at various scan rates, (b) rate capability, and (c) color change performance of the EMS-3 using 1 M LiCl electrolyte with EVB.
